# Application of Moire Profilometry in Three-Dimensional Profile Reconstruction of Key Parts in Railway

**DOI:** 10.3390/s22072498

**Published:** 2022-03-24

**Authors:** Ruyu Ma, Jinlong Li, Kailang He, Tao Tang, Yu Zhang, Xiaorong Gao

**Affiliations:** School of Physical Science & Technology, Southwest Jiaotong University, Chengdu 610031, China; maruyu97@126.com (R.M.); hekailang97@126.com (K.H.); tangtao_swjtu@126.com (T.T.); zhang.yuer@163.com (Y.Z.); gxrr@vip.163.com (X.G.)

**Keywords:** wheel tread, moire profilometry, 3D reconstruction, frequency domain filtering, nondestructive testing

## Abstract

Moire profilometry (MP) is one of the three-dimensional (3D) topography measurement methods of structured light, which has the advantages of single frame reconstruction, high speed, no contact and high precision, and is suitable for dynamic measurement scenes. In this article, the digital MP is applied to the wheel tread measurement, the virtual grating is generated by computer to project to the object surface, the moire fringe pattern of the object is obtained by filtering, and finally the continuous phase pattern is obtained by phase unwrapping. The 3D shape reconstruction of the wheel tread is realized, and a new method of wheel tread detection is provided. At the same time, in this paper, the results of using different filters are compared, and the significance of the frequency domain filtering to MP is proved. It is necessary to choose a suitable filtering method according to different environmental conditions. At present, digital MP can be used in industrial static detection, and it can be extended to the dynamic detection of rolling wheels in the future, so as to improve the detection efficiency and realize the automatic detection of trains.

## 1. Introduction

Optical three-dimensional (3D) measurement based on the grating projection has the advantages of high speed, no contact with the object to be tested, high resolution and so on, so it is suitable for the 3D detection of wheel-rail shape [1,2,3,4,5,6,7,8,9,10]. Phase measurement profilometry (PMP) is one of the commonly used forms of optical projection 3D profilometry, but it is not suitable for real-time or dynamic measurement because it needs to collect multiple grating images, and the movement of the object affects the measurement results. In addition, motion blur of the deformed fringe patterns collected will occur when the object is in a state of high-speed rotation. While the wheels are in a rotating state in many cases, and if a detection method suitable for rolling wheels can be found, the efficiency of detection can be greatly improved. The general method used in real-time measurement is Fourier transform profilometry (FTP), because it only needs to collect one frame of image, the amount of operation is small and the processing speed is high, it is easy to realize the measurement of motion scene. However, it is necessary to ensure that there is no aliasing between all levels of spectrum, so it is difficult to improve the accuracy [11,12].

MP is a non-contact 3D surface measurement method, which superimposes the reference grating and the deformed grating of the object to form moire fringe patterns, and restores the surface profile of the object by calculating the distribution of moire fringe patterns. It can be divided into shadow method, projection method, digital synthesis method and so on [13]. Li et al. proposed a 3D measurement method based on computer-generated moire fringe. It has great potential in real-time and dynamic 3D measurement because of the characteristics of its single launch deformation pattern. This method could avoid color or texture interference and has higher measurement accuracy than FTP [14]. He also proposed a high-precision computer generated MP, based on accurate elimination of background light components [15]. Tang et al. proposed a phase compensation method to solve the problem of 2π ambiguity in moire projection method [16]. Mohammadi Fatemeh et al. calculated multiple moire wavelengths in the full working depth and applied them to the heterodyne multi-wavelength phase unwrapping in digital moire phase unwrapping [17]. Lu Wang et al. proposed a method of computer generated moire profilometry with orthogonal modulation, which reduced the influence of spectrum aliasing and further improved the measurement accuracy of MP [18]. He also proposed a computer-generated moire profilometry based on flat image demodulation, which greatly reduces the influence of frequency aliasing by projecting composite gratings and demodulating [19]. At present, the research of MP is mainly focused on theory and simulation experiments, which is not as widely used as PMP and FTP in engineering. MP has higher precision than FTP and simpler operation than PMP, which greatly improves its practical application value. The requirements for the load and speed of trains are increasing due to the rapid development of railway construction, and the loads on wheels and rails are also increasing, which make people pay more and more attention to the detection of wheels and rails [20]. Wheels are one of the key components of trains, whose safety state is directly determined by their running state. Wheel 3D profile detection is an important means to detect wheel defects and ensure the safety of train operation. The application of MP in wheel profile detection can effectively improve production efficiency.

Manual detection is the most commonly used method of traditional wheel detection, but it has some disadvantages, such as being prone to fatigue, unable to observe directly and so on. The acceleration detection method is often used for detecting the damage under the surface of wheel tread. Zhang et al. put forward an online non-contact method for measuring the wheel tread geometric parameters based on the opto-electronic measurement technique, but it can only measure the size and not restore the shape of the wheel [21]. Zhang applied FTP and wavelet transform profilometry (WTP) to the measurement of 3D topography of wheels, and realized 360°, fast and accurate measurement [22]. Qin et al. applied a new phase-height mapping algorithm when using PMP to detect wheel tread, which is more convenient than the traditional algorithm but cannot realize dynamic measurement [23]. Liu et al. proposed a wheel condition monitoring system based on fiber Bragg grating (FBG), which can detect wheel tread defects online when the train is passing. However, whether the abnormal signal is caused by wheel defects or not needs further analysis [24].

A 3D profile detection method of wheel tread based on MP is presented in this paper. First of all, the sinusoidal grating fringe patterns are generated by computer and projected onto the wheel tread surface. In the experiment, only one frame of deformed grating fringe pattern of the tread is collected, and the reference fringe pattern and deformed fringe pattern are processed by computer, such as frequency domain filtering, to generate the moire fringe pattern of the wheel tread. Then the 3D reconstruction of wheel tread is obtained by phase unwrapping and phase-height mapping. This method can meet the needs of real-time or online measurement for train wheel tread. Experimental results show that the reconstruction errors of MP and PMP are similar, and the 3D shape of the wheel can be reconstructed well. In addition, it has a good performance in defect recognition without changing the projection grating frequency.

## 2. Principle

**Figure 1:**
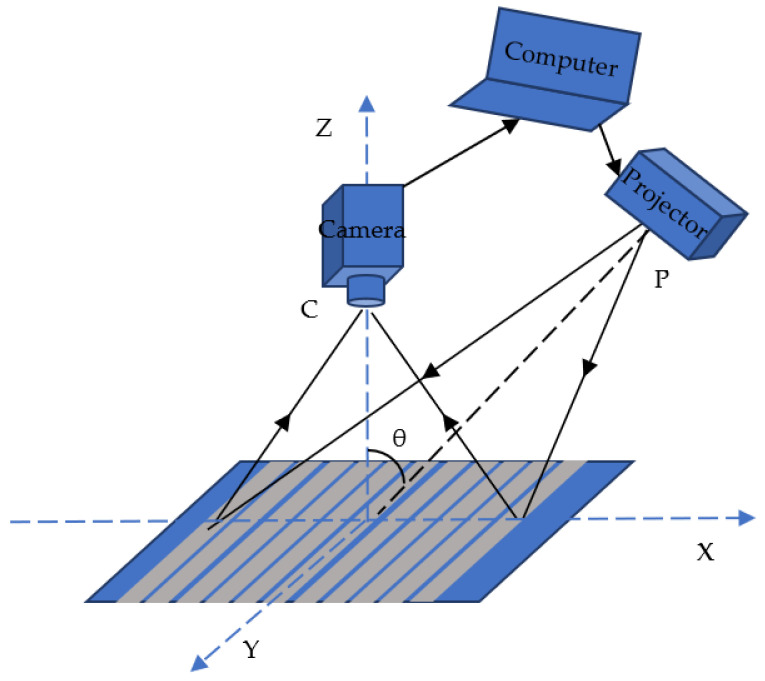
3D reconstruction measurement optical system of MP.

The optical system for 3D reconstruction of object surface based on MP is shown in Figure 1. The optical axis PO of the projector intersects with the optical axis CO of camera at the O point, the angle is θ in the XOZ plane. The computer is used for controlling the projector to project a sinusoidal grating fringe pattern on the surface of the object, taking the surface of the load table as the reference plane. The height of the object will cause the stripe to deform in the X direction and produce the deformed fringe modulated by the height of the object.

The transmittance of one digital sinusoidal fringe pattern can be expressed as:

(1) $$I^{\prime }\left(x^{\prime },y^{\prime }\right)=a+b\cos\left(2\pi fx^{\prime }\right)$$

where, $\left(x^{\prime },y^{\prime }\right)$ represents the image coordinate system, *a* and *b* are constant, and f is the fringe frequency.

Four sinusoidal grating fringe patterns with a phase difference of π/2 are generated by computer, and the digital fringe pattern is projected to the reference plane by the projector, that is, the height of the object h(x,y)=0. The deformed fringe pattern modulated by the reference plane collected by camera can be expressed in the following equations:

(2) $$I_{1}\left(x,y\right)=R\left(x,y\right)\left[A\left(x,y\right)+B\left(x,y\right)\cdot \cos\varphi_{0}\left(x,y\right)\right]$$

(3) $$I_{2}\left(x,y\right)=R\left(x,y\right)\left\{A\left(x,y\right)+B\left(x,y\right)\cdot \cos[\varphi_{0}\left(x,y\right)+\pi /2]\right\}$$

(4) $$I_{3}\left(x,y\right)=R\left(x,y\right)\left\{A\left(x,y\right)+B\left(x,y\right)\cdot \cos[\varphi_{0}\left(x,y\right)+\pi ]\right\}$$

(5) $$I_{4}\left(x,y\right)=R\left(x,y\right)\left\{A\left(x,y\right)+B\left(x,y\right)\cdot \cos[\varphi_{0}\left(x,y\right)+3\pi /2]\right\}$$

In these equations, (x,y) represents the camera system coordinate system, R(x,y) represents the reflectivity distribution of the reference plane, A(x,y) represents the intensity of the background light, B(x,y) represents the contrast of the fringe, $\varphi_{0}\left(x,y\right)=2\pi f_{0}x+\phi_{0}\left(x,y\right)$ is the phase distribution of the deformed fringe pattern on the reference plane, $f_{0}$ is the fundamental frequency of the collected fringe pattern, and $\phi_{0}\left(x,y\right)$ is the phase modulation on the reference plane. The DC component R(x,y) A(x,y) can be eliminated with the help of two fringe patterns with π phase difference. The DC component of $I_{1}\left(x,y\right)$ is eliminated by 180° phase shift sinusoidal grating fringe pattern, and the DC component of $I_{2}\left(x,y\right)$ is eliminated by 270° phase shift sinusoidal grating fringe pattern. The AC components of $I_{1}\left(x,y\right)$ and $I_{2}\left(x,y\right)$ can be expressed by Equations (6) and (7):

(6) $${\tilde{I}}_{1}^{R}\left(x,y\right)=\left[I_{1}\left(x,y\right)-I_{3}\left(x,y\right)\right]/2=R\left(x,y\right)B\left(x,y\right)\cos\varphi_{0}\left(x,y\right)$$

(7) $${\tilde{I}}_{2}^{R}\left(x,y\right)=\left[I_{2}\left(x,y\right)-I_{4}\left(x,y\right)\right]/2=R\left(x,y\right)B\left(x,y\right)\cos[\varphi_{0}\left(x,y\right)+\pi /2]$$

A digital sinusoidal grating fringe pattern is projected on the surface of the measured object, the deformed fringe pattern modulated by the height of the object obtained by camera can be expressed by Equation (8):

(8) $$I_{o}\left(x,y\right)=R^{\prime }\left(x,y\right)\left[A\left(x,y\right)+B\left(x,y\right)\cdot \cos\varphi \left(x,y\right)\right]$$

where, $R^{\prime }\left(x,y\right)$ represents the reflectivity distribution of the object surface, $\varphi \left(x,y\right)=2\pi f_{0}x+\phi \left(x,y\right)$ represents the phase distribution of the deformed fringe on the object surface, and ϕ(x,y) is the phase modulation produced by the measured object and the reference plane. Transform the deformed fringe to frequency domain, and the AC component of the deformed fringe pattern is obtained after the DC component passing the filter.

(9) $${\tilde{I}}_{o}^{O}\left(x,y\right)=R^{\prime }\left(x,y\right)B\left(x,y\right)\cdot \cos\varphi \left(x,y\right)=I_{o}\left(x,y\right)-abs\left(FFT^{-1}\left\{FFT\left\{I_{o}\left(x,y\right)\right\}H\left(u,v\right)\right\}\right)$$

where, H(u,v) represents the function of the filter. By multiplying the obtained AC component ${\tilde{I}}_{o}^{O}\left(x,y\right)$ with the 0° and 90° phase shift sinusoidal grating fringe pattern AC components ${\tilde{I}}_{1}^{R}\left(x,y\right)$ and ${\tilde{I}}_{2}^{R}\left(x,y\right)$, respectively, which can be expressed by Equations (10) and (11):

(10) $$\begin{array}{ll} I_{1}^{OR}\left(x,y\right)= & {\tilde{I}}_{o}^{O}\left(x,y\right)\times {\tilde{I}}_{1}^{R}\left(x,y\right)=R\left(x,y\right)R^{\prime }\left(x,y\right)B^{2}\left(x,y\right)\cos\varphi_{0}\left(x,y\right)\cos\varphi \left(x,y\right)\\ & =R\left(x,y\right)R^{\prime }\left(x,y\right)B^{2}\left(x,y\right)\cos\left[\varphi \left(x,y\right)+\varphi_{0}\left(x,y\right)\right]/2\\ & +R\left(x,y\right)R^{\prime }\left(x,y\right)B^{2}\left(x,y\right)\cos\left[\phi \left(x,y\right)-\phi_{0}\left(x,y\right)\right]/2 \end{array}$$

(11) $$\begin{array}{ll} I_{2}^{OR}\left(x,y\right)= & {\tilde{I}}_{o}^{O}\left(x,y\right)\times {\tilde{I}}_{2}^{R}\left(x,y\right)=R\left(x,y\right)R^{\prime }\left(x,y\right)B^{2}\left(x,y\right)\cos[\varphi_{0}\left(x,y\right)+\pi /2]\cos\varphi \left(x,y\right)\\ & =-R\left(x,y\right)R^{\prime }\left(x,y\right)B^{2}\left(x,y\right)\sin\left[\varphi \left(x,y\right)+\varphi_{0}\left(x,y\right)\right]/2\\ & +R\left(x,y\right)R^{\prime }\left(x,y\right)B^{2}\left(x,y\right)\sin\left[\phi \left(x,y\right)-\phi_{0}\left(x,y\right)\right]/2 \end{array}$$

The second term in Equations (10) and (11) eliminate the phase information of the reference plane and contain the phase information of the object. In order to acquire moire fringe patterns, it is necessary to use frequency domain filtering to extract the second term in Equations (10) and (11). Transform them to the frequency domain and extract them by the filter. The results obtained are recorded as moire fringe pattern 1 and moire fringe pattern 2, respectively, which are expressed as Equations (12) and (13):

(12) $$\begin{array}{ll} I_{moire1}\left(x,y\right)= & R\left(x,y\right)R^{\prime }\left(x,y\right)B^{2}\left(x,y\right)\cos\left[\phi \left(x,y\right)-\phi_{0}\left(x,y\right)\right]/2\\ & =FFT^{-1}\left\{FFT\left\{I_{1}^{OR}\left(x,y\right)\right\}H\left(u,v\right)\right\} \end{array}$$

(13) $$\begin{array}{ll} I_{moire2}\left(x,y\right)= & R\left(x,y\right)R^{\prime }\left(x,y\right)B^{2}\left(x,y\right)\sin\left[\phi \left(x,y\right)-\phi_{0}\left(x,y\right)\right]/2\\ & =FFT^{-1}\left\{FFT\left\{I_{2}^{OR}\left(x,y\right)\right\}H\left(u,v\right)\right\} \end{array}$$

where, H(u,v) represents the function of the low-pass filter used for extracting moire fringe patterns. The phase distribution modulated by the height of the object can be expressed as Equation (14):

(14) $$\phi \left(x,y\right)-\phi_{0}\left(x,y\right)=\arctan[\frac{I_{moire2}\left(x,y\right)}{I_{moire1}\left(x,y\right)}]$$

**Figure 2:**
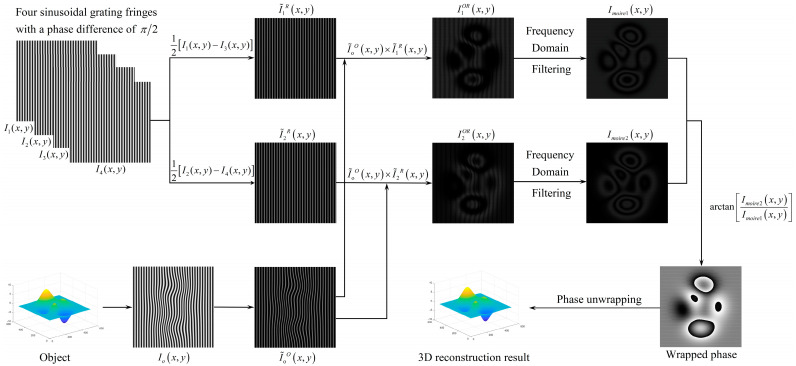
3D reconstruction of object surface based on MP.

The phase calculated by Equation (14) is within the interval [−π,π] range of the inverse trigonometric function, rather than a continuous value, while the phase distribution corresponding to the height distribution of the object is continuous. The phase unwrapping method can restore the wrapped phase to the continuous one, and realize the 3D reconstruction of the object through the phase-height mapping relation.

(15) $$\frac{1}{h\left(x,y\right)}=a\left(x,y\right)+b\left(x,y\right)\frac{1}{\psi \left(x,y\right)}+c\left(x,y\right)\frac{1}{\psi^{2}\left(x,y\right)}$$

where, h(x,y) represents the height of the object and ψ(x,y) represents the continuous value of the phase change caused by the height of the object. The values of a(x,y), b(x,y) and c(x,y) can be obtained by measuring three planes of known height and then solving the system of equations or by reconstructing more known planes then using the least square method to solve. The flow chart of the specific steps is shown in Figure 2.

## 3. Simulation Experiment and Analysis

### 3.1. Simulation

Use the simulation software to build the peaks function as the object to be tested. The simulation software used in this paper is MATLAB, and the peaks function used can be expressed as Equation (16):

(16) $$z=3{\left(1-x\right)}^{2}e^{-x^{2}-{\left(y+1\right)}^{2}}-10(\frac{1}{5}x-x^{3}-y^{5})e^{-x^{2}-y^{2}}-\frac{1}{3}e^{-{\left(x+1\right)}^{2}-y^{2}}$$

**Figure 3:**
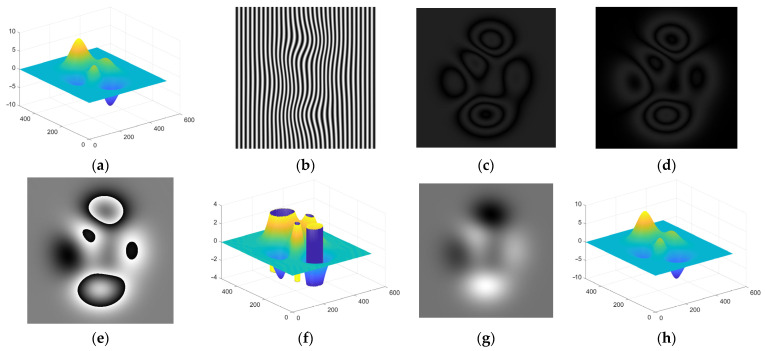
Simulation experiment results of peaks. (**a**) object; (**b**) deformed fringe pattern; (**c**) moire fringe pattern 1; (**d**) moire fringe pattern 2; (**e**) wrapped phase; (**f**) wrapped phase reconstruction result; (**g**) unwrapped phase; (**h**) unwrapped phase reconstruction result.

Gaussian matrix can be generated by using peaks function, as shown in Figure 3a. In the simulation experiment, the grating is projected onto the surface of the object to get a deformed fringe pattern, as shown in Figure 3b. The size of the projection grating is 512 × 512 pixels, and the period p_0_ is 16 pixels. The moire fringe pattern 1 and 2 shown in Figure 3c,d can be obtained according to the flow chart of Figure 2. Then the wrapped phase modulated by the height of the object and its reconstruction results are acquired, as shown in Figure 3e,f. Finally, after phase unwrapping, the continuous phase is obtained as shown in Figure 3g, and the 3D reconstruction of the original object is realized, as shown in Figure 3h.

**Figure 4:**
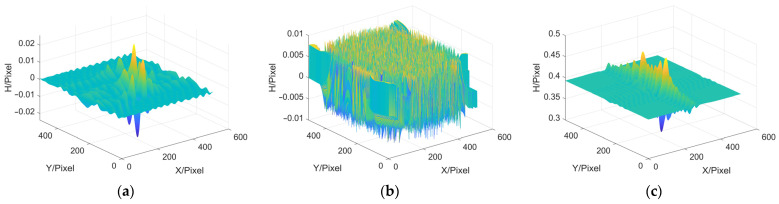
Error distributions. (**a**) MP; (**b**) PMP; (**c**) FTP.

**Figure 5:**
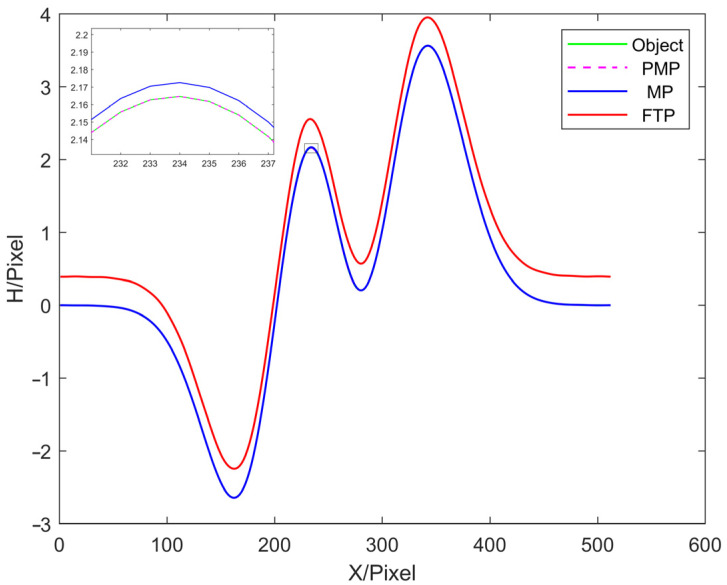
The cutaway of the 250th column.

In this paper, three commonly used 3D reconstruction methods are selected for simulation experiment comparison, which are the MP, PMP and FTP methods. The error distributions between the reconstruction results and the object to be tested are shown in Figure 4. Then, the height of the 250th column of the object to be tested and the reconstruction results are compared, as shown the cutaway views in Figure 5. The experimental results are shown in Table 1.

In terms of reconstruction results, the accuracy of PMP is the highest, and the accuracy of MP and FTP is lower than that of PMP due to the need for frequency domain filtering. However, unlike FTP, the accuracy of MP is only slightly lower than that of PMP. It can also reconstruct the 3D shape of the object accurately, indicating that it still has good detection ability even with the characteristics of single-frame reconstruction.

### 3.2. Frequency Domain Filtering

**Figure 6:**
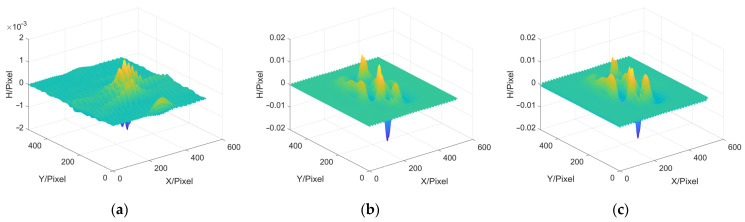

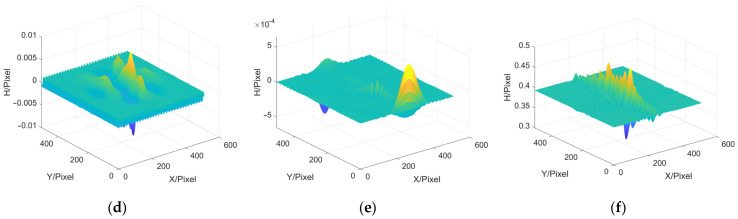
Reconstruction errors. (**a**) MP based on Ideal low-pass; (**b**) MP based on Hanning; (**c**) MP based on Blackman; (**d**) MP based on Kaiser; (**e**) MP based on Hilbert transform; (**f**) FTP.

Next, we discuss the influence of filter window on the accuracy of MP. In this paper, ideal low-pass filter, Hanning window, Blackman window, Kaiser window and Hilbert transform are selected and applied to the step of extracting moire fringe in MP, and the MP reconstruction error results of each filtering method are acquired. In addition, the reconstruction error results obtained by the FTP method, which also belongs to single-frame measurement, are displayed and used to compare the reconstruction accuracy of the FTP method and the MP method as a whole. The error results are shown in Figure 6 and Table 2.

In Table 2, $\bar{h}$ represents the average height, while MAX, MAE and RMSE represent the maximum error, the average absolute error and the root mean square error, respectively. In addition, the average height of the peaks model generated by the simulation software is 0.22192. From the above results, it can be seen that in the step of extracting moire fringe by MP method, the error of using the Blackman window is the largest and that of using the Hilbert transform is the smallest. And no matter which filtering method is used, the reconstruction result of the MP method is better than that of the FTP method.

Under this noise-free simulation condition, the MP method has higher accuracy than the same single-frame method FTP. Furthermore, in the MP method, if we perform one Hilbert transform to a signal, taking the opposite number of the result, and perform another Hilbert transform to the new result, the DC component of the original signal could be well filtered, so as to get satisfied results.

**Figure 7:**
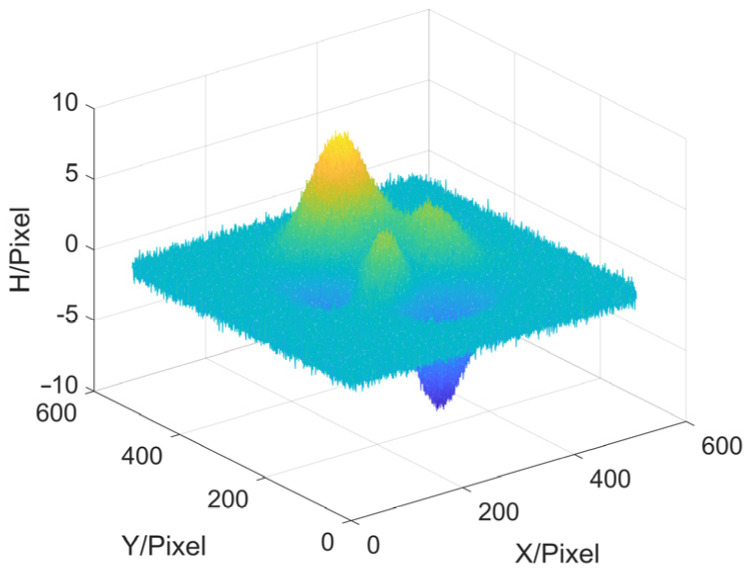
Object with noise to be tested.

**Figure 8:**
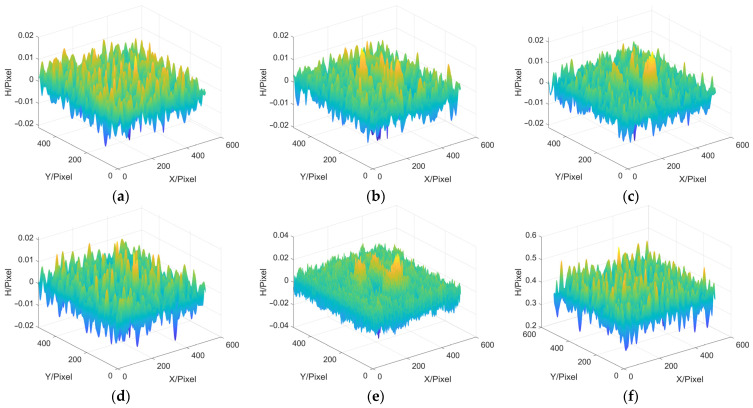
Reconstruction errors (With noise). (**a**) MP based on Ideal low-pass; (**b**) MP based on Hanning; (**c**) MP based on Blackman; (**d**) MP based on Kaiser; (**e**) MP based on Hilbert transform; (**f**) FTP.

Next, we try to add noise to the object to prove the anti-noise performance of MP method. The noise added is a 512 × 512 normal distribution random number matrix with a standard deviation of 0.4, and the object with noise to be tested is shown in Figure 7. The error results and data of the simulation are shown in Figure 8 and Table 3.

The average height of the object with noise to be tested is 0.22192, too. It can be seen that, different from the previous conclusion, when the captured images contain a lot of noise, the results obtained by the MP method with different filters are similar, but the results are still better than that of the FTP.

**Figure 9:**
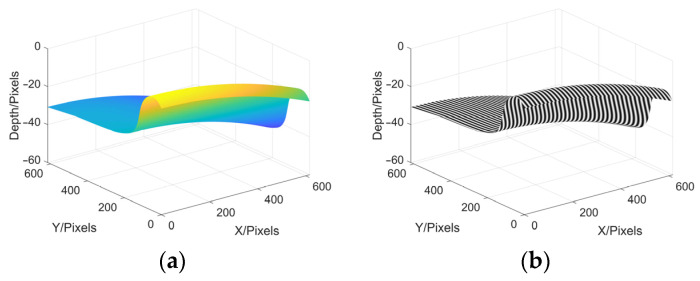
Create wheel tread and project sinusoidal grating fringe pattern. (**a**) wheel tread to be tested; (**b**) projection.

In order to verify whether MP is also suitable for railway wheel measurement, a wheel tread is then created as an object to be tested, and one sinusoidal grating fringe pattern is projected onto its surface, as shown in Figure 9.

**Figure 10:**
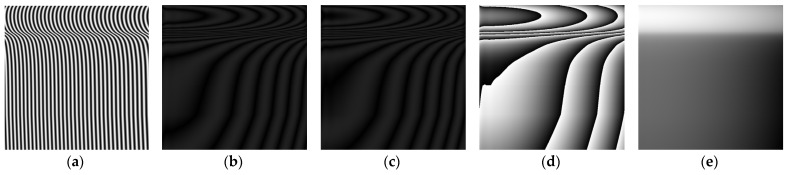
Simulation process of wheel tread reconstruction. (**a**) deformed fringe; (**b**) moire fringe pattern 1; (**c**) moire fringe pattern 2; (**d**) wrapped phase; (**e**) continuous phase.

Following the steps of MP, deformed fringe pattern, moire fringe pattern 1, moire fringe pattern 2, wrapped phase and continuous phase are obtained, as shown in (a), (b), (c), (d) and (e) in Figure 10, respectively.

**Figure 11:**
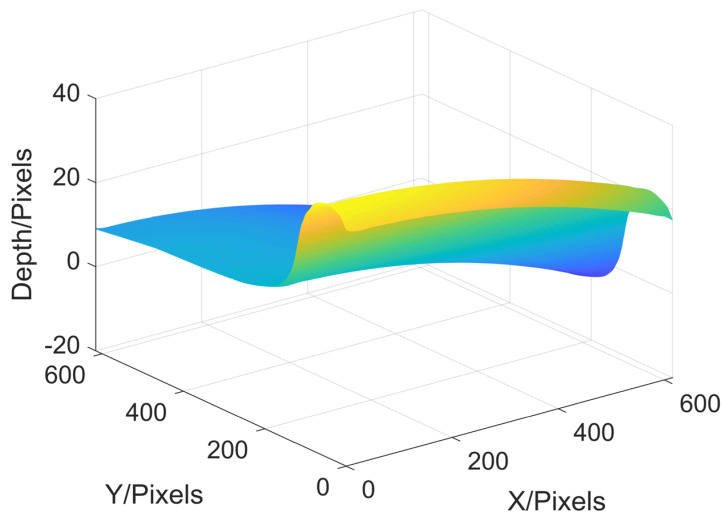
Reconstruction results of wheel tread simulation.

The final reconstruction result of the wheel tread is shown in Figure 11, and the 3D shape of the original wheel tread can be seen clearly. This simulation shows that MP can not only reconstruct peaks function, but can also be suitable for the measurement of railway wheels.

### 3.3. Experiment

In order to further prove the effectiveness and application of digital MP, it is applied to wheel tread detection.

**Figure 12:**
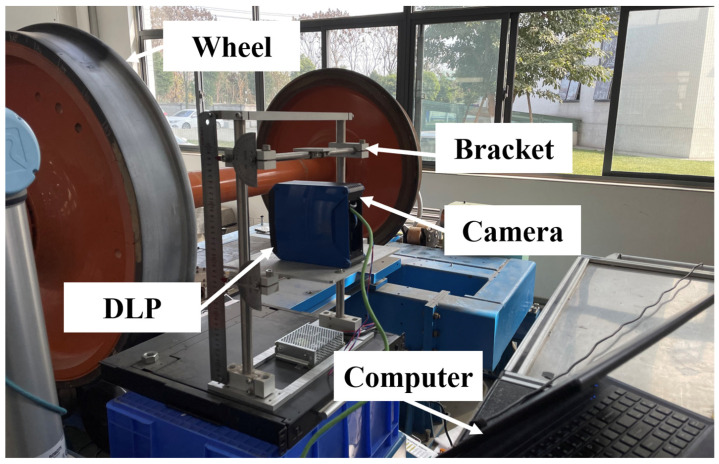
Wheel tread detection experimental platform.

**Figure 13:**
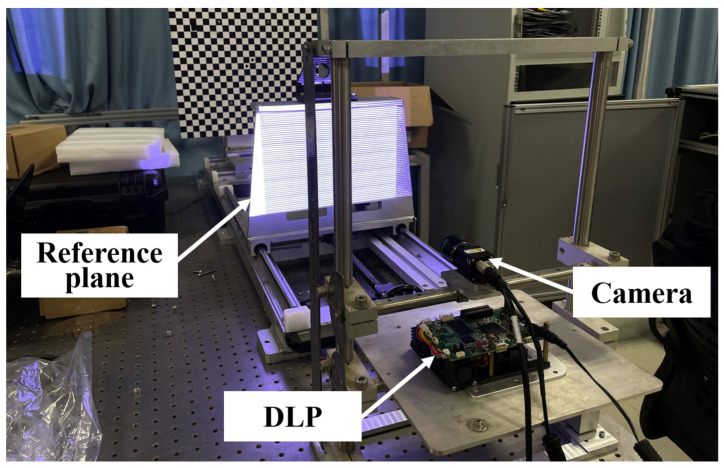
Calibration platform.

First of all, the wheel tread experiment platform needs to be built, as shown in Figure 12. The experimental platform is composed of CMOS camera, digital light processing (DLP) projector, computer, bracket, railway wheel and so on. The railway wheel is the object to be tested in this experiment, and the MP will be used to reconstruct the 3D profile of the wheel tread. The model of the CMOS camera used in this experiment is Basler acA1920-40gm with the resolution of 1200 × 1920 pixels and the model of the DLP is LightCrafter4500. Before the wheel tread detection experiment, the experimental system needs to be calibrated in order to obtain the phase-height mapping relationship, and the calibration platform is shown in Figure 13.

**Figure 14:**
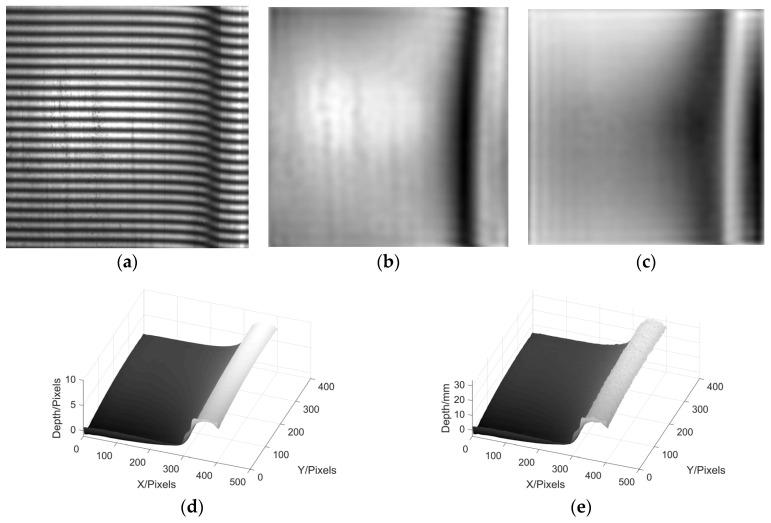
Experimental reconstruction results of wheel tread. (**a**) deformed fringe; (**b**) moire fringe pattern 1; (**c**) moire fringe pattern 2; (**d**) phase reconstruction; (**e**) height reconstruction.

Next, four sinusoidal grating fringe patterns with a phase difference of π/2 are generated. The fringe spatial period p_0_ is 16 pixels, which is projected to the reference plane and collected by CMOS camera. The AC component is obtained by calculating and processing the patterns, so as to eliminate the influence of background light. Then a sinusoidal fringe pattern is selected and projected to the wheel tread, and the deformation pattern modulated by the wheel tread is collected by the CMOS camera. The result is shown in Figure 14a. According to the flow of Figure 2, the moire fringe patterns 1 and 2 are obtained respectively as shown in Figure 14b,c. After calculating the wrapped phase and phase unwrapping, the 3D reconstruction results of the wheel tread are obtained.

It can be seen from Figure 14d, the 3D shape of the wheel tread has been basically restored by using MP, while Figure 14e is the result of wheel tread height reconstruction based on phase-height mapping relationship after the calibration.

**Figure 15:**
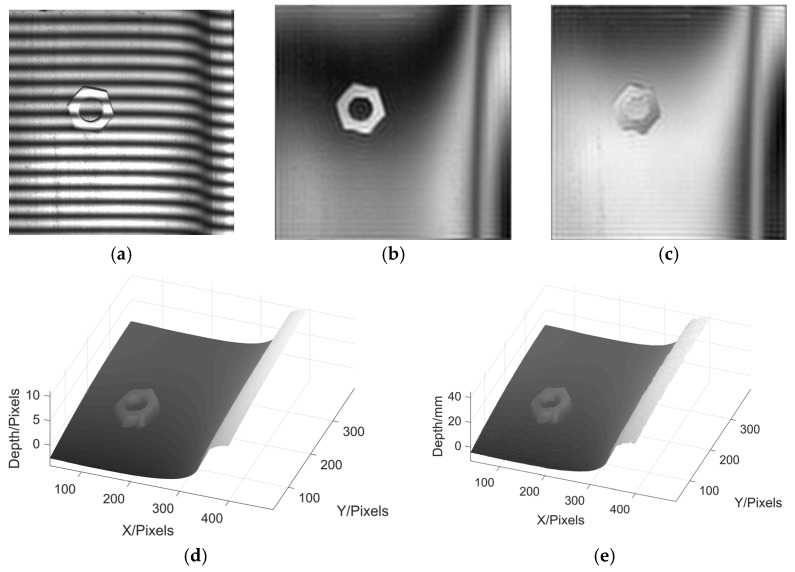
Experimental reconstruction results of wheel tread with a nut. (**a**) deformed fringe; (**b**) moire fringe pattern 1; (**c**) moire fringe pattern 2; (**d**) phase reconstruction; (**e**) height reconstruction.

Lastly, we paste a nut on the surface of the wheel and measure it by MP again. This is used to test the ability of MP to measure discontinuous height. The experimental results are shown in Figure 15.

From the above results, it can be seen that MP has a good ability to reconstruct the 3D profile of wheel tread. It not only has a good effect in measuring the wheel tread, but also can clearly restore the morphology of the nut on the tread without changing the grating frequency. 

**Figure 16:**
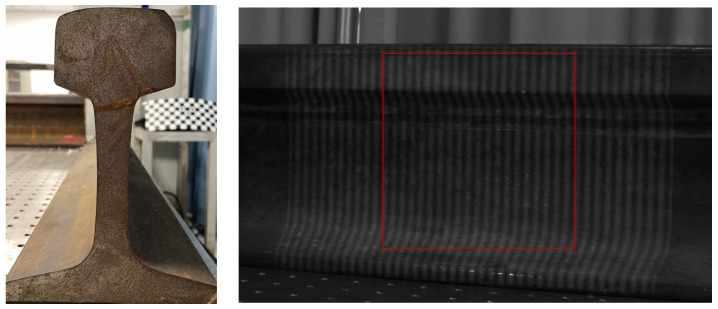
Rail shape and deformed fringe pattern.

**Figure 17:**
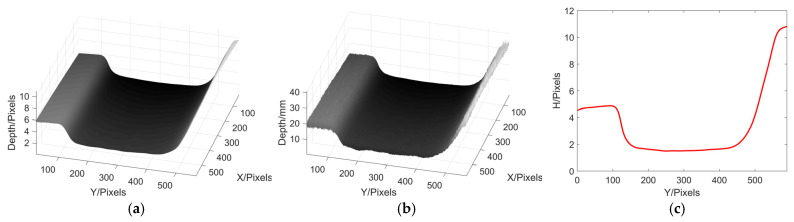
Experimental reconstruction results of rail. (**a**) phase reconstruction; (**b**) height reconstruction; (**c**) The cutaway of the 300th row.

Except for reconstructing the 3D profile of the wheel tread, we also used the MP to reconstruct the rail. The shape of the rail and the projected grating fringe pattern are shown in Figure 16, and the 3D profile reconstruction result of the rail is shown in Figure 17.

From the above result, it can be seen that the 3D profile of the rail can be well reconstruction by using MP. It is proved that the application of MP in industry is feasible.

**Figure 18:**
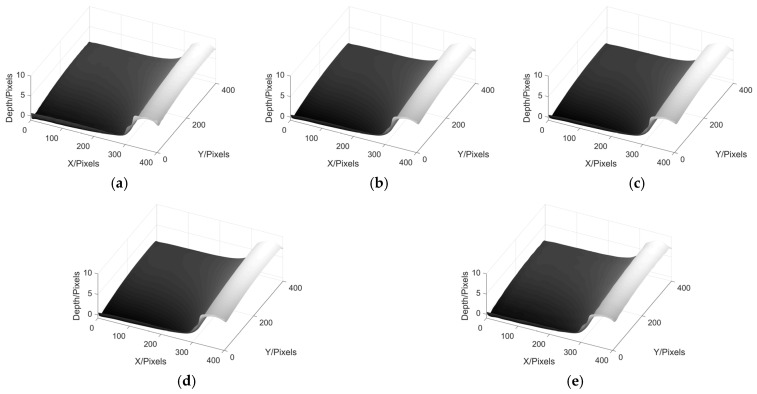
Phase reconstruction results of wheel tread using different filters. (**a**) MP based on Ideal low-pass; (**b**) MP based on Hanning; (**c**) MP based on Blackman; (**d**) MP based on Kaiser; (**e**) MP based on Hilbert transform.

**Figure 19:**
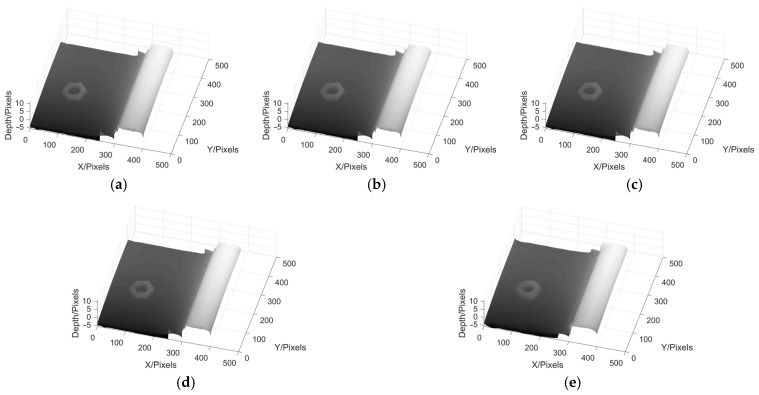
Phase reconstruction results of wheel tread with a nut using different filters. (**a**) MP based on Ideal low-pass; (**b**) MP based on Hanning; (**c**) MP based on Blackman; (**d**) MP based on Kaiser; (**e**) MP based on Hilbert transform.

Next, we take the wheel tread reconstruction experiment as an example to compare the effects of different filtering methods on phase reconstruction in MP. Figure 18 shows the results of wheel tread phase reconstruction using ideal low-pass filter, Hanning window, Blackman window, Kaiser window and Hilbert converter, while Figure 19 shows the phase reconstruction results of wheel tread with a nut.

From the above results, it can be seen that in the experiment of wheel tread phase reconstruction, the 3D profile of the wheel tread can be well reconstructed by using these five filtering methods. In the experiment of phase reconstruction of wheel tread with a nut, the six corners of the nut can be seen clearly by the first four methods, but the edge of the nut cannot be seen in the result obtained by Hilbert transform. When there are many components in the spectrum of image, it becomes more difficult to use Hilbert transform to remove the DC component from the image. When conducting the MP experiment, it is necessary to choose the most suitable filter according to different conditions in order to obtain satisfactory results.

## 4. Discussion

In this paper, we build a wheel tread detection experimental platform based on railway background. We can capture the wheel tread deformed fringe pattern in real time and the 3D shape of wheel can be reconstructed by MP method. Compared with other 3D measurement methods, MP method is easy to operate and efficient in measurement. 

In the above wheel tread measurement experiment, we find that when the spatial period of the projected sinusoidal grating fringe is too high or too low, the reconstruction effect is not satisfactory. When the spatial period of sinusoidal grating fringe is 16 pixels, the best reconstruction result could be obtained. At the same time, in the process of reconstruction, the selection of filter parameters is also crucial. Thus, the conditions including the spatial period of projection fringes and filter parameters need to be optimized according to different measurement environments. In addition, we know that MP depends on the method of grating fringe patterns projection, so conditions such as extremely strong background light or specular reflection will reduce the fringe contrast, make the collected pictures blurry, and then affect the measurement. Therefore, when using MP for measurement, it is necessary to be in a dark or low-light experimental environment, and the object to be measured should not have specular reflection. If the collected pictures have weak specular reflection, the relevant algorithms can be used to process the pictures and eliminate the impact.

For industrial measurement, safety, efficiency and convenience are often valued, and digital MP can be used in non-destructive testing of wheel tread because of its single frame and non-contact measurement. When the wheel is rolling, the MP method could restore the 360° shape of the wheel tread by stitching, but the processing of the reference background also needs to be further considered. Therefore, the image stitching technology and the processing of the reference background will be the next step.

## 5. Conclusions

In this paper, based on digital MP, three commonly used structured light projection methods are firstly compared through simulation experiments. It can be seen that, compared with the PMP method, the characteristics of the single capture image of MP makes it easier to operate, so it has more advantages in dynamic on-line measurement. While, compared with the single frame reconstruction method FTP, which can also be used for on-line measurement, MP has higher accuracy. In addition, by comparing the simulation experiments of noise-free condition and noise condition, it is concluded that when using MP for measurement, it is necessary to select the appropriate filter window for different measurement scenes in order to obtain the best measurement results.

At the same time, MP is also applied to the 3D profile detection of the wheel tread in this paper, and the 3D shape of the wheel tread is successfully reconstructed with a good effect. Compared with the traditional MP measurement method, this kind of digital MP is easier to operate and has higher efficiency and accuracy, which provides a new method for on-line non-contact detection of wheel tread. This technology can be used in wheel-rail 3D profile reconstruction and measurement, and can be expanded to measure and analyze rolling wheels in the future, while improving the detection efficiency and realizing automatic detection of train wheel and rail.

## Figures and Tables

**Table 1:** Comparison of experimental results.

Methods	MP	PMP	FTP
MAX	0.0275	0.0092	0.4813
RMSE	0.0031	0.0029	0.3942

**Table 2:** Reconstruction errors.

Methods	$\bar{h}$	MAX	MAE	RMSE
Ideal lowpass	0.22192	0.00156	0.00005	0.00012
Hanning	0.22192	0.01391	0.00085	0.00164
Blackman	0.22192	0.01442	0.00119	0.00210
Kaiser	0.22192	0.00889	0.00094	0.00129
Hilbert transform	0.22192	0.00065	0.00001	0.00002
FTP	0.61462	0.47981	0.39270	0.39279

**Table 3:** Reconstruction errors (With noise).

Methods	$\bar{h}$	MAX	MAE	RMSE
Ideal lowpass	0.22211	0.01742	0.00342	0.00430
Hanning	0.22144	0.01943	0.00350	0.00459
Blackman	0.22198	0.02165	0.00344	0.00460
Kaiser	0.22139	0.01726	0.00346	0.00435
Hilbert transform	0.22134	0.03879	0.00355	0.00526
FTP	0.61480	0.56464	0.39288	0.39468

## Data Availability

Not applicable.
